# The Effect of Commercially Available Desensitizers on Bond Strength Following Cementation of Zirconia Crowns Using Self-Adhesive Resin Cement—An In Vitro Study

**DOI:** 10.3390/ma15020514

**Published:** 2022-01-10

**Authors:** Harisha Dewan, Mohammed E. Sayed, Nasser M. Alqahtani, Tariq Alnajai, Abdulaziz Qasir, Hitesh Chohan

**Affiliations:** 1 Department of Prosthetic Dental Sciences, College of Dentistry, Jazan University, Jazan 45142, Saudi Arabia; drsayed203@gmail.com; 2 Department of Prosthetic Dental Sciences, College of Dentistry, King Khalid University, Abha 61421, Saudi Arabia; nmalqahtani@kku.edu.sa; 3 College of Dentistry, Jazan University, Jazan 45142, Saudi Arabia; tariq.alnajai@gmail.com (T.A.); dr.aziz32@gmail.com (A.Q.); 4 Department of Restorative Dental Sciences, College of Dentistry, Jazan University, Jazan 45142, Saudi Arabia; drhiteshchohan@yahoo.co.in

**Keywords:** cementation, bond strength, dentin-desensitizing agents, tooth preparation

## Abstract

The improvement of the tensile strength of zirconia crowns after the application of commercially available desensitizers can provide added advantages for the durability and strength of zirconia prostheses. We assessed the retention of zirconia crowns when Gluma, Shield Force Plus, and Telio CS desensitizers were used with resin luting cement. Four groups with ten specimens each (*n* = 10) were considered as Group 1 (Control group, with no desensitizer application before crown cementation with resin cement) and Groups 2, 3, and 4 (with a single coat of Gluma dentin desensitizer, Telio CS desensitizer, or Shield Force Plus desensitizer applied before crown cementation, respectively). Thermocycling was then carried out, and each group was tested to determine the associated retentive forces and type of failure. The data were statistically analyzed, which showed that the mean tensile-strength values were significantly higher in Group 2 (*p*-value = 0.001), Group 3 (*p*-value = 0.027), and Group 4 (*p*-value = 0.014), when compared with the Control group. Clinicians should consider the application of any of these three desensitizers, as they can successfully abate dentin hypersensitivity after tooth preparation, as well as increase the durability and strength of the zirconia prosthesis.

## 1. Introduction

For prosthetic replacements and the reconstruction of lost crown structures, all-ceramic crowns have become popular for various reasons, such as increased acceptance by patients, esthetics, stability, and biocompatibility [1]. Various types of ceramics are available, including oxides and glass ceramics. These are usually luted to the prepared tooth with a resin cement, due to their ability to chemically adhere to the prepared tooth surface. These resin cements also chemically bond with the ceramic surfaces, thereby strongly holding both the tooth and the crown structure. Less microleakage has also been noted when using these types of cements [2].

In the process of tooth preparation to receive the crown, the loss of the tooth structure leads to the pain and sensitivity, which is the most common complaint of patients either during tooth preparation or after the procedure. This is caused by dentin hypersensitivity, described as a sharp pain that lingers for some time. This pain is usually felt when intaking cold drinks or with the impact of the air or any other stimuli that lead to fluid movement in the dentinal tubules [3]. The preparation of the tooth and the luting cement are both key factors that may have an impact on the dental hypersensitivity [4].

To prevent dental hypersensitivity, the application of dental desensitizing agents is one of the most preferred methods [5]. These dental desensitizing agents may interact with the luting agents, thus potentially altering the retentive properties of the cement [6]. Previous studies have compared various desensitizers for the reduction of sensitivity in prepared teeth [7,8,9,10]. A recent study by Sayed, M.E. in 2021 stated the decrease in post-tooth-preparation dentin sensitivity due to the application of Gluma (Heraeus Kulzer, Hanau, Germany), Shield Force Plus (Tokuyama Dental America Inc., San Diego, CA, USA), and Telio CS (Ivoclar Vivadent, Schaan, Liechtenstein) desensitizing agents [11]. However, the effect of these desensitizing agents on the resin luting cement, in terms of the resulting all-ceramic-crown bond strength, is still unknown. Hence, the aim of the present study was to assess the effect of the application of the above desensitizers before the cementation of zirconia crowns with RelyX U-200 cement (a self-adhesive resin cement). The hypothesis for the study tests considered whether the tensile bond strength of zirconia crowns after using the desensitizers was improved or not after cementing with the self-adhesive resin cement and then thermocycling.

## 2. Materials and Methods

### 2.1. Materials and Groups

The research was initiated after the research protocol was approved by the Institutional Review Board at the College of Dentistry, Jazan University (Reference No. CODJU-19181).

**Figure 1:**
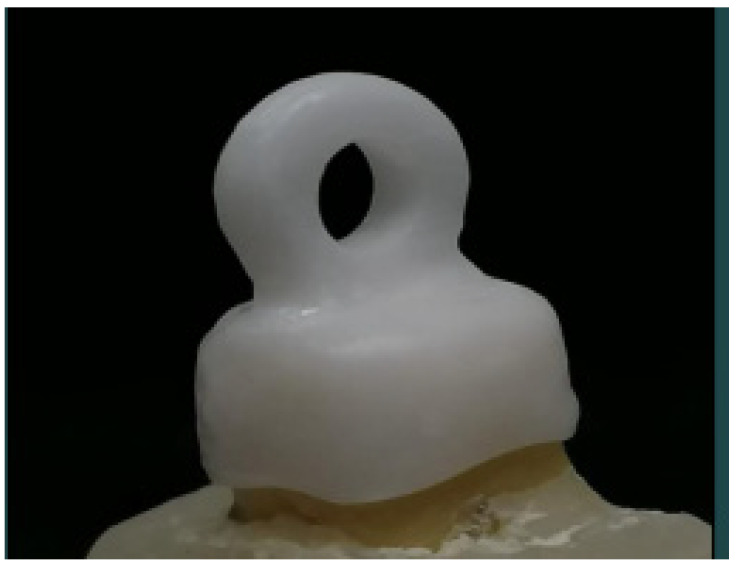
Zirconia coping, designed with a coronal loop (4 mm outer diameter and 2 mm inner diameter) to facilitate tensile loading.

For this study, 40 freshly extracted, non-carious human molars were collected. The selected teeth were not previously restored. The teeth were disinfected with 0.5% sodium hypochlorite solution, debris removed with a scaler, and kept in distilled water. Retentive grooves were made on the roots and poly (methyl methacrylate) resin (Quick resin, Ivoclar, Schaan, Liechtenstein) was used to mount the teeth 1–2 mm below the cementoenamel junction (CEJ). The horizontal arm of a surveyor was customized to securely hold a high-speed handpiece. New diamond burs (C1-Strauss, Ra’anana, Israel) were used, and the standard protocol was followed for tooth preparation (finish line prepared as chamfer, 0.4 mm in width, and located 1 mm above the CEJ; axial walls with height of 4 mm, and a proximal taper of 10°). Dental Wings Open Software (DWOS 8.1, Dental Wings INC.,MTL, Canada) was used to design 40 zirconia copings, 1.5 mm in thickness, with a coronal loop of 4 mm external diameter and 2 mm internal diameter on the occlusal surface, to facilitate tensile testing (Figure 1). The copings were milled (Milling machine Model k5, vhf camfacture AG, Ammerbuch, Germany) in pre-sintered zirconia blanks (Ceramill Zl, Amann Girrbach AG, Koblach, Austria). 

All 40 of the mounted teeth were made to equal samples (i.e., 10 samples each in four groups).

Group 1: Control; no desensitizer was applied, crown cementation performed with resin cement later on.

Group 2: Treated with Gluma dentin desensitizer, crown cementation later performed with resin cement.

Group 3: Treated with Telio CS desensitizer, crown cementation later performed with resin cement.

Group 4: Treated with Shield Force Plus desensitizer, crown cementation later performed with resin cement.

The details of each desensitizer, regarding their group number, trade name, batch number, manufacturer, composition, and mechanism of action, are listed in Table 1.

### 2.2. Desensitizer Application

Moisture was removed and Ivoclean (Ivoclar Vivadent, Schaan, Liechtenstein) was used to clean the intaglio surfaces of the copings. The manufacturer’s instructions were followed, and one coat of the dental desensitizing agent was applied to all the groups (except for the Control group) before cementation.

#### 2.2.1. Gluma Desensitizer Group

An applicator brush was used to coat Gluma desensitizer on the prepared tooth surface and left for 30–60 s. The dentin surface was carefully dried with an air stream, sprayed with plenty of water, and vacuumed.

#### 2.2.2. Telio CS Group

A thin layer of Telio CS desensitizer was applied with an applicator brush and left for 10 s. The excess was dispersed into a thin layer using an air syringe.

#### 2.2.3. Shield Force Plus Group

Shield Force desensitizer was applied with an applicator brush and left untouched for 10 s. The dispensed desensitizer was protected from ambient light using a light-blocking plate. Weak air flow was continuously applied to the desensitizer surface for 5 s, followed by strong air flow for another 5 s. The surface was light cured (intensity > 300 m W/cm^2^) for 10 s, keeping the light-curing tip within a distance of 2 mm.

### 2.3. Cementation

The copings were cemented with Rely X U-200 cement following the manufacturer’s instructions under finger pressure. The sandblasting unit (TJK-BP II, Tianjin Haide, Tianjin, China) with 50-micron aluminum-oxide particles was used to sandblast the intaglio surface of the copings at a pressure of 2 Bar (30 psi). The surface was then cleaned with alcohol and air dried. The prepared tooth was cleaned with pumice paste, rinsed, and lightly dried, leaving the tooth surface moist. A small amount of cement was dispensed onto the mixing pad, in order to ensure a perfect mix, and was discarded. The cement was then directly dispensed into the coping. The coping was firmly seated using finger pressure, and was tack cured for 1–2 s per surface using a light-curing unit (Elipar S10, 3M-ESPE). Excess cement was removed with a scaler while holding the coping in place. A final light cure was carried out for 20 s per surface.

### 2.4. Thermocycling

**Figure 2:**
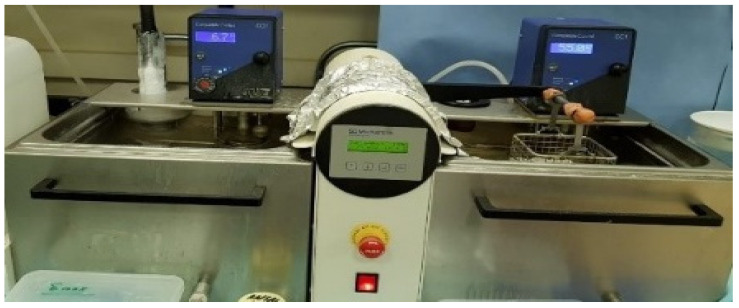
Thermocycling from 5 °C to 55 °C for 3000 cycles using a dwell time of 30 s.

The specimens were then stored in water at 37 °C for 30 days, after which thermocycling from 5 °C to 55 °C for 3000 cycles using a dwell time of 30 s was carried out in a thermocycling unit (Model 1100, SD Mechatronik, Bayern, Germany) (Figure 2). This was performed to simulate normal stresses [12]. 

### 2.5. Testing and SEM Analysis

**Figure 3:**
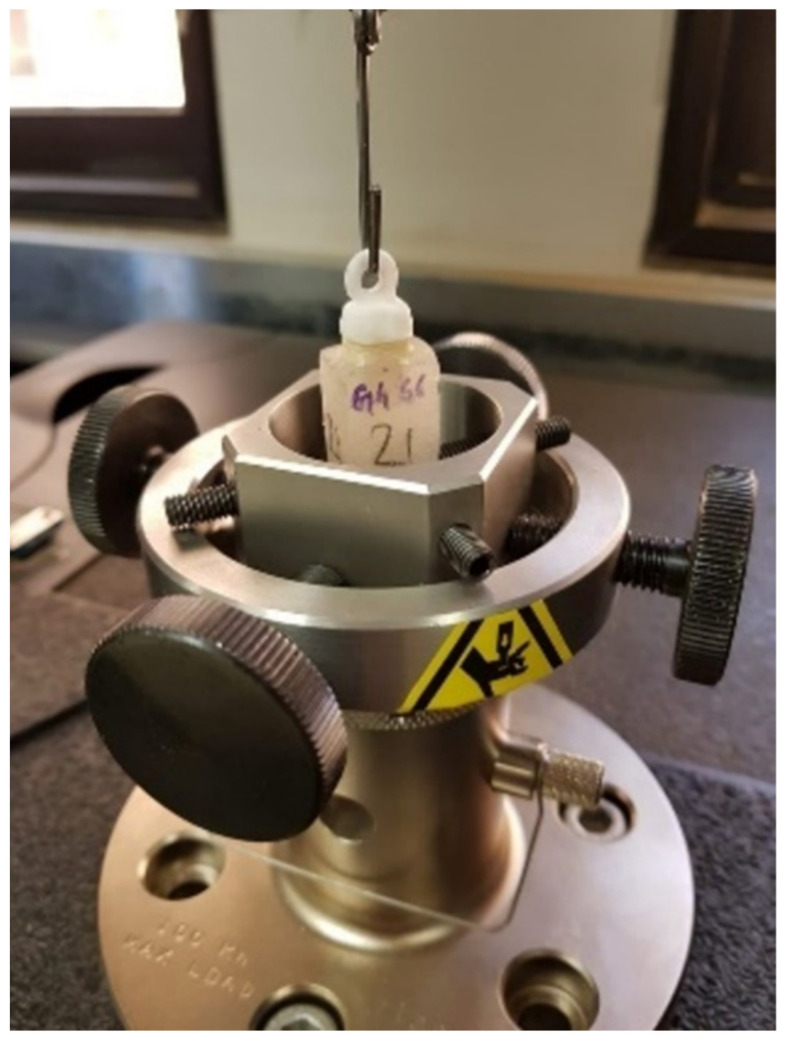
Universal testing machine subjecting copings to dislodgment forces until failure occurred.

A 1.2 mm-diameter metal wire was then hooked through the previously described coronal loop, and the crowns were then subjected to dislodgment forces until failure occurred. A crosshead speed of 1 mm/min was used and testing was carried out using a universal testing machine (Instron, Model 4502, Instron Corp., Buckinghamshire, UK; Figure 3). The maximum load at failure was recorded which, when divided by the total surface area of each preparation, gave the tensile strength value (Table 2). 

**Figure 4:**
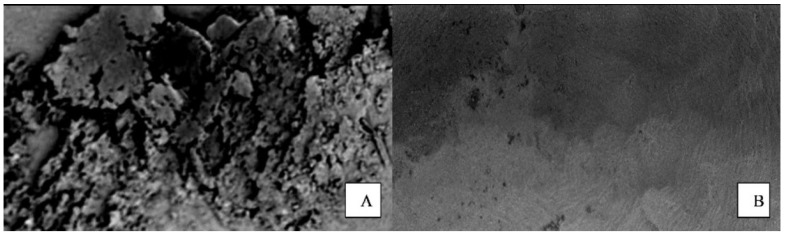
Example of the de-bonded surfaces inspected for failure and its type under 20× magnification (Scanning Electron Microscope, Hitachi High-Tech, HHT, Tokyo, Japan). (**A**). Image of the dentin surface, (**B**). Image of the intaglio surface of the coping.

The de-bonded surfaces were inspected for failure and its type under 20× magnification (Scanning Electron Microscope, Hitachi High-Tech, HHT, Japan; Figure 4). The criteria that were followed to classify the failures are described in Table 3 [11].

### 2.6. Statistical Analysis

The mean and standard deviation (SD) of means for maximum loads and tensile strength (descriptive statistics) were calculated using the Statistical Product and Service Solutions version 15 software (SPSS Inc., Chicago, IL, USA). The maximum loads applied and the tensile strengths in Groups 1–4 are shown in Table 1. To establish that the four groups followed normal distribution, the Shapiro–Wilk test was applied. Levene’s Statistic Test of Homogeneity of Variance was carried out to assess the equality of variances. Multiple group comparisons were carried out by One-way ANOVA, and the pairwise comparisons were conducted using the post hoc Bonferroni test. Statistical analyses with *p*-values less than 5% were considered statistically significant. The failure type distribution between the four groups was analyzed using the chi-square test.

## 3. Results

The distribution and variance of data were analyzed before using One-way ANOVA. Shapiro–Wilk test clearly stated that the values of tensile strengths in the four groups followed a normal distribution (Table 4). 

The Test of Homogeneity of Variances by Levene’s Statistic stated a significant difference between the variances of tensile strength values in the four groups with a *p*-value of 0.001 (Table 5). It was inferred that the variances were different in the four groups.

**Figure 5:**
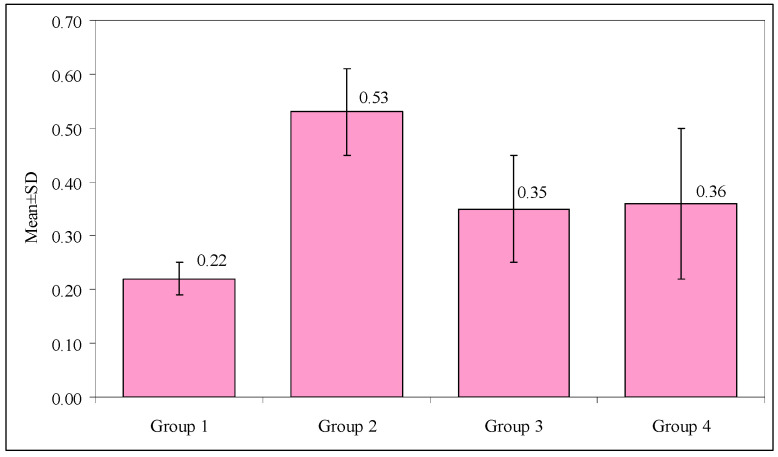
Comparison of mean values of the tensile strengths in Groups 1–4. Group 1: Control; Group 2: Treated with Gluma dentin desensitizer; Group 3: treated with Telio CS desensitizer; Group 4: Treated with Shield Force Plus desensitizer.

The mean values for the tensile strengths, when compared between the four groups (Group 1 = 0.22 MPa, Group 2 = 0.53 MPa, Group 3 = 0.35 MPa, and Group 4 = 0.36 MPa) using the One-way ANOVA test, are shown in Table 6. Their *p*-values were all less than 0.001, indicating statistical significance. Figure 5 shows the comparison of the mean values of the tensile strengths in the four groups.

The inter-group comparison of the mean values for the tensile strengths was conducted using the post hoc Bonferroni test (Table 7). The mean value was significantly higher among Group 2 (*p*-value = 0.001), Group 3 (*p*-value = 0.027), and Group 4 (*p*-value = 0.014), when compared with that of the Control group. There were significant differences when Group 2 was compared with Groups 3 and 4, with *p*-values of 0.001 and 0.003, respectively. However, the difference in the mean values between Groups 3 and 4 was not significant.

**Figure 6:**
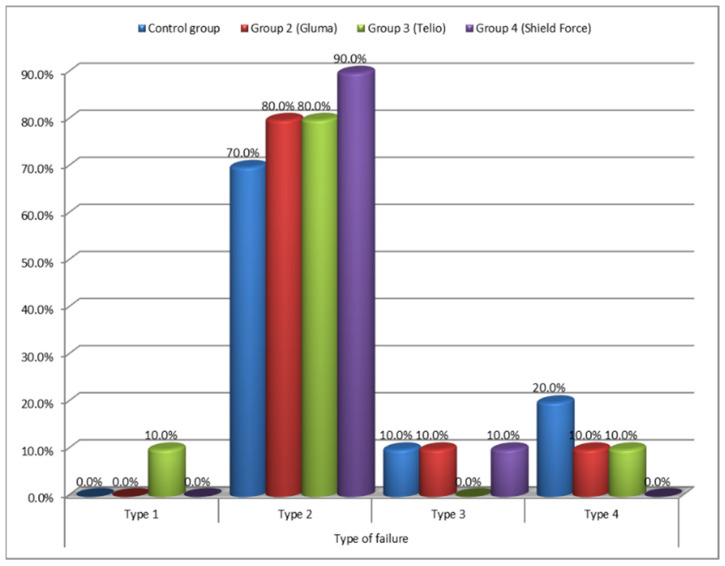
Comparison of the failure-type distribution in the four groups. Group 2: Treated with Gluma dentin desensitizer; Group 3: Treated with Telio CS desensitizer; Group 4: Treated with Shield Force Plus desensitizer.

Figure 6 shows the failure-type distribution when comparing the four groups using the chi-square test. The most common failure was Type 2, with 80% occurrence in Groups 2 and 3, 70% in Group 1, and 90% in Group 4. The distribution of the type of failure between the four groups was statistically insignificant (chi-square = 6.2502, *p-*value= 0.7150).

## 4. Discussion

Desensitizing agents are commonly applied in order to control pain, thus making dental procedures much more comfortable for patients that require fixed dental prostheses [13,14,15,16,17,18,19,20]. The Gluma, Shield Force Plus, and Telio CS desensitizers effectively decrease the post-preparation sensitivity, as has been shown in the study conducted in 2021 by Sayed, M.E. [11]. The same desensitizers were chosen in the present study in order to evaluate their effect on the strength of the bond to the prepared tooth when using self-adhesive resin cement. The three agents showed varying retentive values: Gluma being the most effective, followed by Shield Force Plus, and then Telio CS. This difference could be due to the differences in their components or their mode of action, their capability to resist dissolution, and the different solubility level of precipitate formation in the dentinal tubules [11]. The hydrophilic properties provided by Hydroxyethyl methacrylate (HEMA) in the Gluma and Shield Force Plus desensitizers improved the bonding to the hydrophilic dentin. A condensation reaction takes place between HEMA (in the desensitizers) and phosphate (in the self-adhesive resin cement) with the elimination of water, leading to a better bond [11]. In the present study, we observed the greatest tensile strength in the Gluma group. Similar observations were made in the study of Chandravarkar, S.M. and others [14,16,17,18,19,20,21,22].

Resin is a common luting agent for all-ceramic crowns. Luting agents are chiefly applied for adhesion with the dentin and the prevention of microleakage. In the study of Wolfart, it was stated that, when Gluma was applied, the abutment surface was similar to the dentine as observed under the microscope [15]. Reinhardt, in 1995, stated that the Gluma desensitizer did not affect the bond strength of resin luting agents [16]. Similar observations have been made in the study by Mausner [17].

The study observed no statistical differences in the occurrence of various types of fractures within the four groups. However, Type 2 failure was the most common for all the fractures observed, which corresponded to the cement being present on both the coping and tooth, indicating a cohesive failure within the cement. Similar observations have been made in the study of Jalandar, S.S. [18]. A possible reason for this could be that the bond strength of the desensitizers and dentin, and between the desensitizers and self-adhesive resin cement, was higher than that of the bond strength of the self-adhesive resin cement itself [23]. This failure can be considered favorable in regard to the present study as the increase in bond strength caused by the application of desensitizers may have led to the obvious results. However, Asadullah (2018) observed that, for all-cast-metal crowns, the type of fracture was of the adhesive type when Gluma was used [19].

An unexpected finding was the occurrence of failure within the dentin (coronal or root-fracture type 4) in two samples of the Control, and one sample each of Gluma and Telio CS Groups. This indicated the higher tensile strength of the tooth-coping assemblies than the inner strength of the tooth [11]. Adhesive failure in the interface of the dentin and cement (Type 1 failure) was seen in only one sample of the Telio CS Group. Type 3 failure (at the interface of the zirconia coping and cement) was also found only in one sample each of the Control, Gluma and Shield Force Plus Groups. These findings are in concordance with the studies showing adequate bond strength of the self-adhesive cement with zirconia and dentin [24,25,26].

Our findings provide a clear indication that the desensitization of dentin with all three of the considered desensitizers resulted in the improved tensile strength of the self-adhesive resin cement after thermocycling; therefore, the hypothesis was accepted.

It should be noted that there were a few limitations to the study. The present study was an in vitro study using a pull-off test with a standard protocol for preparing the human teeth, for applying the desensitizers, and for cementing the crowns; however, the results may vary in the in vivo conditions, when factors such as saliva and other dislodgment forces due to the various textures of foods are involved. The bond strength of resin cement varies with the varied micromorphology of the dentin of the extracted teeth that were used for the study [26]. Furthermore, these extracted molars may have lost some of the dentin-fluid protein, which would have affected the reaction between the desensitizers and the protein. Finally, the present study subjected the specimens to artificial aging through thermocycling, wherein the temperature of all the groups was standardized and the clinical situation was simulated; however, the availability of more longitudinal clinical-aging data could lead to more precise results.

## 5. Conclusions

The results obtained in this study allowed us to conclude that a single application of any of the three desensitizers that were considered before cementation increased the bond strength with a zirconia crown, with the greatest effect being seen with Gluma, followed by Shield Force Plus and Telio CS. There were no major differences in the failure type observed when using the three desensitizing agents or the control.

## Figures and Tables

**Table 1:** Commercial names and details of desensitizers used.

Group	Material Trade Name	Batch No.	Manufacturer	Composition	Mechanism of Action
2	Gluma dentin desensitizer	K010514	Heraeus Kulzer, Hanau, Germany	5% glutaraldehyde and 35% 2-hydroxyethyl methacrylate (HEMA) in an aqueous solution.	Glutaraldehyde and the dentinal proteins react to form precipitates which reduce the tubule diameters.
3	Telio CS desensitizer	Y09693	Ivoclar Vivadent, Schaan, Liechtenstein	35% polyethylene glycol dimethacrylate, 50% glutaraldehyde, 55% water, <0.01% maleic acid.	Polyethylene glycol dimethacrylate (PEG-DMA), along with glutaraldehyde, provides optimal sealing of the tubules.
4	Shield Force Plus desensitizer	140E48	Tokuyama Dental America Inc., San Diego, CA, USA	10–30% 2-hydroxyethyl methacrylate (HEMA), 10–30% bisphenol A dis (2-hydroxy propoxy) dimethacrylate, 10–30% phosphoric acid monomer, 30–60% propan-2-ol, 5–10% triethylene glycol dimethacrylate, 5–10% water.	The adhesive monomer reacts with calcium in the tooth to form the first block. A durable second coating is formed by curing.

**Table 2:** The maximum loads and tensile strengths in Groups 1–4.

Group 1			Group 2			Group 3			Group 4		
S No.	Maximum Load (N)	Tensile Strength (MPa)	S No.	Maximum Load (N)	Tensile Strength (MPa)	S No.	Maximum Load (N)	Tensile Strength (MPa)	S No.	Maximum Load (N)	Tensile Strength (MPa)
1	18.22	0.24	1	48.39	0.64	1	18.78	0.25	1	10.66	0.14
2	20.93	0.27	2	35.05	0.46	2	21.67	0.28	2	22.04	0.29
3	16.83	0.22	3	32.25	0.42	3	13.90	0.18	3	43.10	0.57
4	15.52	0.20	4	45.4	0.60	4	20.47	0.27	4	35.71	0.47
5	13.02	0.17	5	37.36	0.49	5	30.65	0.40	5	25.37	0.33
6	19.18	0.25	6	44.80	0.59	6	34.95	0.46	6	15.26	0.20
7	16.11	0.21	7	30.82	0.40	7	27.17	0.36	7	17.20	0.23
8	17.85	0.23	8	42.47	0.56	8	36.27	0.48	8	29.45	0.39
9	14.34	0.19	9	44.67	0.59	9	29.74	0.39	9	34.92	0.46
10	13.92	0.18	10	38.34	0.50	10	31.30	0.41	10	38.37	0.50

**Table 3:** Failure classification criteria.

Classification	Description	Criteria
Type 1	Most of the cement present on the intaglio surface of the coping	Adhesive failure (cement–dentin interface)
Type 2	Cement present both on dentin and intaglio surface of the coping	Cohesive failure (within cement)
Type 3	Most of the cement present on the dentin surface	Adhesive failure (cement–crown interface)
Type 4	Coronal or root fracture	Cohesive failure (within dentin)

**Table 4:** Normality test of tensile strength scores in four groups by Shapiro–Wilk test.

Variables	Groups	Statistic	Degree of Freedom	*p*-Value
Tensile strength	Group 1	0.9810	10	0.9700
	Group 2	0.9360	10	0.5110
	Group 3	0.9480	10	0.6470
	Group 4	0.9630	10	0.8150

Group 1: Control; Group 2: Treated with Gluma dentin desensitizer; Group 3: Treated with Telio CS desensitizer; Group 4: Treated with Shield Force Plus desensitizer.

**Table 5:** Results of Test of Homogeneity of Variances by Levene’s Statistic.

Variables	Levene Statistic	Degree of Freedom 1	Degree of Freedom 2	*p*-Value
Tensile strength	7.5210	3	36	0.001 *

* Significant difference (*p* < 0.05).

**Table 6:** Comparison of the mean values of tensile strengths in Groups 1–4 using One-way ANOVA.

	Mean (MPa)	Standard Deviation	Minimum (MPa)	Maximum (MPa)	F-Value	*p*-Value
Group 1	0.22	0.03	0.17	0.27	16.935	<0.001 *
Group 2	0.53	0.08	0.40	0.64		
Group 3	0.35	0.10	0.18	0.48		
Group 4	0.36	0.14	0.14	0.57		

* Significant difference (*p* < 0.05). Group 1: Control; Group 2: Treated with Gluma dentin desensitizer; Group 3: Treated with Telio CS desensitizer; Group 4: Treated with Shield Force Plus desensitizer.

**Table 7:** Inter-group comparison of the mean values of the tensile strengths using the post hoc Bonferroni test.

First Group	Second Group	Mean Difference (MPa)	*p*-Value
Group 1	Group 2	−0.31	0.001 *
Group 1	Group 3	−0.13	0.027 *
Group 1	Group 4	−0.14	0.014 *
Group 2	Group 3	0.18	0.001 *
Group 2	Group 4	0.17	0.003 *
Group 3	Group 4	−0.01	1.000

* Significant difference (*p* < 0.05). Group 1: Control; Group 2: Treated with Gluma dentin desensitizer; Group 3: Treated with Telio CS desensitizer; Group 4: Treated with Shield Force Plus desensitizer.

## Data Availability

The data that support the findings of this study are available from the corresponding author upon reasonable request.

## References

[1] Stawarczyk B., Hartmann L., Hartmann R., Roos M., Ender A., Ozcan M., Sailer I., Hämmerle C.H.F. (2012). Impact of Gluma Desensitizer on the tensile strength of zirconia crowns bonded to dentin: An in vitro study. Clin. Oral Investig..

[2] Behr M., Rosentritt M., Regnet T., Lang R., Handel G. (2004). Marginal adaptation in dentin of a self-adhesive universal resin cement compared with well-tried systems. Dent. Mater..

[3] Addy M. (1990). Etiology and clinical implications of dentine hypersensitivity. Dent. Clin. N. Am..

[4] Kern M., Kleimeier B., Schaller H.G., Strub J.R. (1996). Clinical comparison of postoperative sensitivity for a glass ionomer and a zinc phosphate luting cement. J. Prosthet. Dent..

[5] Erdemir U., Yildiz E., Kilic I., Yucel T., Ozel S. (2010). The efficacy of three desensitizing agents used to treat dentin hypersensitivity. J. Am. Dent. Assoc..

[6] Yim N.H., Rueggeberg F.A., Caughman W.F., Gardner F.M., Pashley D.H. (2000). Effect of dentin desensitizers and cementing agents on retention of full crowns using standardized crown preparations. J. Prosthet. Dent..

[7] Watanabe T., Sano M., Itoh K., Wakumoto S. (1991). The effects of primers on the sensitivity of dentin. Dent. Mater..

[8] Dondi dall’Orologio G., Malferrari S. (1993). Desensitizing effects of Gluma and Gluma 2000 on hypersensitive dentin. Am. J. Dent..

[9] Felton D., Bergenholtz G., Kanoy B.E. (1991). Evaluation of the desensitizing effect of Gluma dentin bond on teeth prepared for complete-coverage restorations. Int. J. Prosthodont..

[10] Bergenholtz G., Jontell M., Tuttle A., Knutsson G. (1993). Inhibition of serum albumin flux across exposed dentine following conditioning with GLUMA primer, glutaraldehyde or potassium oxalates. J. Dent..

[11] Sayed M.E., Dewan H., Alomer N., Alsubaie S., Chohan H. (2021). Efficacy of desensitizers in reducing post-preparation sensitivity prior to a fixed dental prosthesis: A randomized controlled clinical trial. J. Int. Soc. Prevent. Communit. Dent..

[12] Gale M.S., Darvell B.W. (1999). Thermal cycling procedures for laboratory testing of dental restorations. J. Dent..

[13] Magne P., So W.S., Cascione D. (2007). Immediate dentin sealing supports delayed restoration placement. J. Prosthet. Dent..

[14] Chandavarkar S.M., Ram S.M. (2015). A comparative evaluation of the effect of dentin desensitizers on the retention of complete cast metal crowns. Contemp. Clin. Dent..

[15] Wolfart S., Linnemann J., Kern M. (2003). Crown retention with use of different sealing systems on prepared dentine. J. Oral Rehabil..

[16] Reinhardt J.W., Stephens N.H., Fortin D. (1995). Effect of gluma desensitization on dentin bond strength. Am. J. Dent..

[17] Mausner I.K., Goldstein G.R., Georgescu M. (1996). Effect of two dentinal desensitizing agents on retention of complete cast coping using four cements. J. Prosthet. Dent..

[18] Jalandar S.S., Pandharinath D.S., Arun K., Smita V. (2012). Comparison of effect of desensitizing agents on the retention of crowns cemented with luting agents: An in vitro study. J. Adv. Prosthodont..

[19] Syed Asadullah S.R., Rakhewar P., Mapkar M.A. (2018). Comparison of effect of desensitizing agents on the retention of crowns cemented with resinomer cement: An in vitro study. Int. J. Prev. Clin. Dent. Res..

[20] Lawaf S., Jalalian E., Roshan R., Azizi A. (2016). Effect of GLUMA desensitizer on the retention of full metal crowns cemented with Rely X U200 self-adhesive cement. J. Adv. Prosthodont..

[21] Reddy S.M., Vijitha D., Deepak T., Balasubramanian R., Satish A. (2014). Evaluation of shear bond strength of zirconia bonded to dentin after various surface treatments of zirconia. J. Indian Prosthodont. Soc..

[22] Sailer I., Tettamanti S., Stawarczyk B., Fischer J., Hämmerle C.H. (2010). In vitro study of the influence of dentin desensitizing and sealing on the shear bond strength of two universal resin cements. J. Adhes. Dent..

[23] Palacios R.P., Johnson G.H., Phillips K.M., Raigrodski A.J. (2006). Retention of zirconium oxide ceramic crowns with three types of cement. J. Prosthet. Dent..

[24] Ernst C.P., Cohnen U., Stender E., Willershausen B. (2005). In vitro retentive strength of zirconia oxide ceramic crowns using different luting agents. J. Prosthet. Dent..

[25] Heintze S.D. (2010). Crown pull-off test (crown retention test) to evaluate the bonding effectiveness of luting agents. Dent. Mater..

[26] Oilo G. (1993). Bond strength testing—What does it mean. Int. Dent. J..

